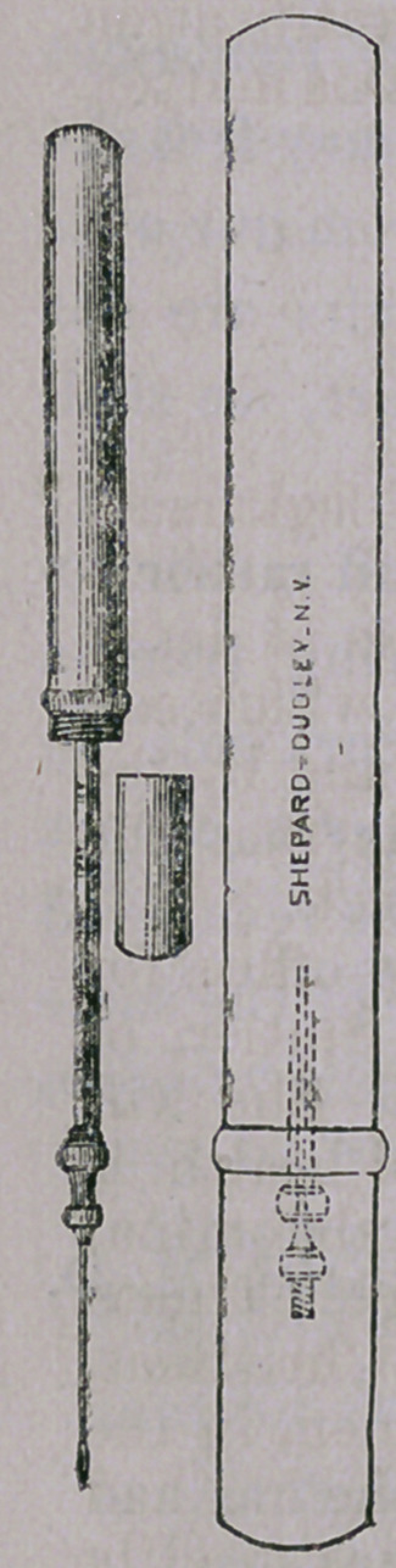# A New Hypodermic Syringe

**Published:** 1873-12

**Authors:** Ephraim Cutter

**Affiliations:** Woburn, Mass.


### MISCELLANEOUS.

### A New Hypodermic Syringe.

BY EPHRAIM CUTTER, M. D., WOBURN, MASS.

Although hypodermic medication has not superseded the gastric, still, for its efficiency, promptness, and energy, it ranks as one of the great advances in modern medicine. It is apprehended that with the mass of the profession it is employed mainly when medication by the stomach fails, and in urgent cases only. Be this as it may, in my own experience I have found that whenever I desired and most needed my hypodermic syringe, it was generally left at home, because the bulk of the box containing it (small as it is) was still found to be cumbersome. This being the case, the writer has sought to contrive a form of syringe which should be so compact as to be carried in the pocket-case of medicines, and occupy the space usually allotted to a phial. The accompanying figures represent in full size the result of this endeavor.

One cut shows the outline of the full-sized instrument, closed. Dotted lines represent the internal arrangement. The other figure illustrates the same with syringe filled, piston drawn out and needle attached. The third figure is a representation of the cap that covers the needle to protect it from harm. The points in which this syringe differs from the ordinary ones are :—

(*a.*) Closure of the distal end of the cylinder. This makes the syringe a *cul-de-sac.* The circulation of the air is quite different from that in the cylinder of the ordinary syringe. Instead of the air drying the leather of the piston on both sides, it only dries on one side—thus reducing the chances of drying fifty per cent. Practically this syringe has kept in good order for months continually, when the ordinary syringe would be loose and dry. The drop of moisture confined in the *cul-de-sac* behind the piston has no connection with the air except through the piston ; hence it evaporates slowly. Another reason for closing the distal end of the syringe was to keep out dirt and foreign substances.

(*b.*) Making the piston and its head hollow throughout, and having a male screw and milled head on the proximal end. By these means a communication is effected with the cavity of the syringe.

(*c.*) Having the needle attached to the proximal end of the piston. This latter arrangement is *the* feature which is novel and which constitutes its peculiarity. The instrument is thus complete and ready for use. It is only necessary to draw out the piston with the needle inserted into the medicament. The fluid readily fills the barrel by running through the needle and piston, and the syringe is charged for use.

The writer is greatly indebted to the firm of Shepard & Dudley, 150 William street, New York City,—the makers of the instrument,—for suggesting the new alumina alloy as a material for the needle. Steel gold-plated needles rust when kept in this instrument, but this difficulty is overcome by the new alloy, which will not rust or tarnish.

The firm mentioned above supply two forms of this instrument; one of German silver, nickel-plated, with alumina alloy needle, price two dollars; and the other entirely of the alloy, price three dollars.

In closing, the writer would say that he prefers extemporaneous solution of morphous salts for subcutaneous injections. The bottom of a glass goblet or tumbler, turned upside down, furnishes a clean and handy cavity in which to deposit the salt. The syringe may be filled with water and its contents discharged on the salt. The circulating motion thus given aids in dissolving the salt, and then it can be taken up into the syringe for use.

If those who use this syringe experience the same gratification as the writer, it will be an ample reward for his pains in this matter. *Boston Journal Chemistry.*

## Figures and Tables

**Figure f1:**